# Optimization of the chicken chorioallantoic membrane assay as reliable *in vivo* model for the analysis of osteosarcoma

**DOI:** 10.1371/journal.pone.0215312

**Published:** 2019-04-15

**Authors:** Pierre Kunz, Astrid Schenker, Heiner Sähr, Burkhard Lehner, Jörg Fellenberg

**Affiliations:** 1 Center for Orthopedics, Trauma Surgery and Paraplegiology, University of Heidelberg, Baden-Württemberg, Heidelberg, Germany; 2 Clinic for Shoulder and Elbow Surgery, Catholic Hospital Mainz, Rhineland-Pfalz, Mainz, Germany; Universita degli Studi di Bari Aldo Moro, ITALY

## Abstract

Survival rates of osteosarcoma patients could not be significantly improved by conventional chemotherapeutic treatment regimens since the introduction of high-dose chemotherapy 35 years ago. Therefore, there is a strong clinical need for new therapeutic targets and personalized treatment strategies, requiring reliable *in vivo* model systems for the identification and testing of potential new treatment approaches. Conventional *in vivo* rodent experiments face ethical issues, are time consuming and costly, being of particular relevance in orphan diseases like osteosarcoma. An attractive alternative to such animal experiments is the chicken chorioallantoic membrane (CAM) assay. The CAM is a highly vascularized, non-innervated extra-embryonic membrane that is perfectly suited for the engraftment of tumor cells. However, only few reports are available for osteosarcoma and reported data are inconsistent. Therefore, the aim of this study was the adaptation and optimization of the CAM assay for its application in osteosarcoma research. Tumor take rates and volumes of osteosarcoma that developed on the CAM were analyzed after modification of several experimental parameters, including egg windowing, CAM pretreatment, inoculation technique and many more. Eight osteosarcoma cell lines were investigated. Our optimized OS-CAM-assay was finally validated against a rat animal xenograft model. Using the cell line MNNG HOS as reference we could improve the tumor take rates from 51% to 94%, the viability of the embryos from initially 40% to >80% and achieved a threefold increase of the tumor volumes. We were able to generate solid tumors from all eight osteosarcoma cell lines used in this study and could reproduce results that were obtained using an osteosarcoma rat animal model. The CAM assay can bridge the gap between *in vitro* cell culture and *in vivo* animal experiments. As reliable *in vivo* model for osteosarcoma research the optimized CAM assay may speed up preclinical data collection and simplifies research on potential new agents towards personalized treatment strategies. Further, in accordance with Russell’s and Burch’s “Principles of Humane Experimental Technique” the reasonable use of this model provides a refinement by minimizing pain and suffering of animals and supports a considerable reduction and/or replacement of animal experiments.

## Introduction

Osteosarcoma is the most common primary tumor of bone in children and young adults [1, 2]. It is characterized by a strong vascularization, cellular heterogeneity, aggressive local tumor growth and early metastasis, mainly into the lungs. Since the introduction of neoadjuvant chemotherapy about 35 years ago, the survival rate for osteosarcoma patients stagnates at approximately 60% and only 20% - 30% for patients with metastases [3–5]. Whereas personalized treatment has been translated into the clinical routine for multiple malignancies, such therapeutic approaches do not exist for osteosarcoma patients so far. The latest and largest multinational therapy-optimizing study “Euramos-1”, evaluating a multi-drug chemotherapy regimen, could not contribute to an improved survival for osteosarcoma patients [6]. Results of the following treatment-optimizing study for osteosarcoma patients won`t be available for more than another decade, emphasizing the clinical need for new diagnostic and therapeutic approaches to improve osteosarcoma patient survival. However, their identification is substantially hampered by the limited availability of appropriate *in vivo* models. In contrast to *in vitro* models they mimic the physiological cancer environment and allow the interaction of tumor cells with the tumor-promoting stroma including endothelial cells and fibroblasts. They further allow the investigation of individualized approaches that are especially important for the treatment of such heterogeneous tumors like osteosarcoma. Yet, current animal models are time consuming, expensive and have provoked ethical concerns. In an orphan disease like osteosarcoma, where research is largely academic driven and not by pharmaceutical companies, these aspects further limit progress of new therapeutic approaches. The chicken chorioallantoic membrane (CAM) assay might be an attractive alternative to conventional animal models. The CAM assay is a well-established and highly reproducible model in the field of angiogenesis [7] and is also widely utilized as an *in vivo* system to study the aggressiveness of various tumors including prostate cancer [8], glioblastoma [9, 10] and colon carcinoma [11]. During the chicken embryo development, the CAM is formed by fusion of the mesodermal layer of the allantois with the mesodermal layer of the chorion. It is highly vascularized, making it an ideal substrate for the cultivation of tumors and the study of angiogenesis. Together with the extracellular matrix proteins (ECM) the CAM mimics the physiological cancer environment. Grafting of tumors is further facilitated by a natural immunodeficiency of the CAM lacking cell-mediated immunity until day 14 [12]. Tumor cells rapidly form three dimensional tumors, infiltrate the surrounding tissue and even metastasize to different organs of the embryo. The CAM is not innervated so that experiments are not associated with pain perception by the embryo and there is no need for ethical approval for animal experimentation. Further advantages of the CAM assay are high reproducibility, cost effectiveness and short incubation times [13]. However, the CAM assay has several methodological challenges when used as an *in vivo* model in tumor research, e.g. inconsistent chick embryo viability, inconsistent and low tumor take rates and delayed or slow tumor growth. Despite these challenges, the CAM assay could be successfully adapted for different tumor entities and is now widely applied for the analysis of several tumors including neuroblastoma [14], squamous cell carcinoma [15], multiple myeloma [16] and glioblastoma [17]. Unfortunately, this adaptation could not yet be achieved for osteosarcoma, resulting in a rare application of this promising *in vivo* model in osteosarcoma research. A PubMed search on osteosarcoma and CAM assays retrieved only 16 matches with conflicting data and little information concerning the achieved tumor take rates, tumor volumes and viability of the embryos. In addition, there are very few reports with detailed protocols [18, 19], none of them presenting a systematic adaptation and optimization of the CAM assay for osteosarcoma, as described for other entities.

Consequently, the aim of our study was the development of a reliable and reproducible *in vivo* xenograft model by systematic adaptation of the CAM assay for its use in osteosarcoma research. We investigated and optimized numerous assay parameters leading to significant improvements of chicken embryo viability, tumor take rates and tumor volumes. In addition, we could successfully validate our final OS-CAM-assay by the comparison of outcome parameters obtained after transplantation of osteosarcoma cells to the CAM with the *in vivo* growth observed in an osteosarcoma rat animal model.

## Materials and methods

### Animal studies

All animal studies were performed in compliance with the national laws relating to the conduct of animal experimentation, were approved by the Animal Experimentation Committee Karlsruhe, Germany (G-144/06) and were performed according to the national guidelines for animal care in accordance with the European Union Directive. Cell suspensions (100000cells/animal) of wild-type, sFLT1 and ANG2 overexpressing UMR-106 osteosarcoma cells were inoculated subcutaneously into the thigh of male RNU (immunodeficient nude) rats (Charles Rivers Laboratories, Sulzfeld, Germany) at an age of 6–8 weeks and an average weight of 195 grams (six animals per group, randomly allocated). Tumor cell inoculation was conducted in inhalation anesthesia, no further painful intervention requiring pain medication were conducted before euthanasia. Daily monitoring minimized distress and suffering, as suspicious rats were identified in an early stage and according to the installed human endpoints euthanized if necessary. Animals were housed in Macrolon type IV cages with one companion. They had unlimited access to food and water and were kept at an 12h light dark cycle at 22°C. Humane endpoints were in place for euthanizing animals. Animals would have been euthanized in case of significantly reduced body weight or body condition (palpation of bones at the iliosacral region). Furthermore, in case of any signs of severe illness like apathy or pain behavior like aggressivity while palpating the tumor, in case of significantly reduced food or water intake, respiratory distress or motoric abnormalities like paralysis, further if the tumor reached the maximum tumor size threshold of 3cm in diameter, in case of an ulceration of the tumor or 80 days after tumor inoculation, whatever occurred first. The maximum body weight loss threshold for euthanizing an animal was 20% of the expected body weight according to RNU growth charts, corrected for estimated tumor weight. No body weight loss was observed over time after tumor inoculation. All animals continuously gained body weight after tumor inoculation in accordance to RNU rat growth charts and independent from the estimated tumor weight. None of the animals showed any severe signs of illness following tumor formation. None of the animals died due to the experimental procedure (tumor cell inoculation) and no unexpected deaths were observed. Two animals showed a beginning ulceration at day 25 after inoculation and where therefore euthanatized according to the defined endpoints. Tumor volumes were measured using calipers as previously described [20]. After subcutaneous inoculation, cumulative tumor volume increased to 44.157mm^3^ for wildtype tumors, 18.371mm^3^ for sFLT-1 tumors and 9.285mm^3^ for Ang-2 tumors at day 25.

### Cell lines and cell culture

The following commercially available osteosarcoma cell line were purchased and used in this study. HOS (#CRL-1543) (American Tissue Culture Collection (ATCC), Rockville, Maryland, USA), MG63 (#CRL-1427) (ATCC), HOS 143B (#91112502), (Sigma-Aldrich, München, Germany), CAL-72 (#ACC-439) (DSMZ, Braunschweig, Germany), U2OS (#300364), (Cell Line Service GmbH, Eppelheim, Germany), MNNG-HOS (#300289) (CLS), Saos-2 (#300331) (CLS) and UMR-106 (#CRL-1661) (ATCC). All cell lines were cultured in Dulbecco’s Modified Eagle Medium high glucose (DMEM) (Biozym, Hessisch Oldendorf, Germany) containing 4.5g/l glucose and supplemented with 10% fetal calf serum (FCS) (Biochrom, Berlin, Germany), and 100U/ml penicillin / streptomycin (Lonza GmbH, Köln, Germany).

### Transfection experiments

For stable overexpression of *sFLT1* and *ANG2* the rat osteosarcoma cell line UMR-106 was transfected with the vector pCMV3 containing the complete coding sequence of either *sFLT1*or *ANG2* (Hölzel Diagnostika, Köln, Germany). Transfection was carried out using the electroporation unit MP-100 (PeqLab, Erlangen, Germany). Cells were cultured until they reached ~80% confluence, trypsinized and washed twice in PBS. For transfection, 10^6^ cells were resuspended in 100 μl R-buffer containing 5μg plasmid DNA. After electroporation with two pulses at 1200 V for 20 ms cells were plated in DMEM-medium and cultured for 72 h before hygromycin (300 μg/ml) (Carl-Roth GmbH) was added to select stable cell lines. Overexpression of *sFLT1* and *ANG2* was validated by quantitative RT-PCR.

### ALU and CR1 in situ hybridization

Digoxigenin-labeled probes for human-specific ALU repetitive DNA sequences and chicken specific CR1 repetitive elements were prepared by PCR containing 1×PCR buffer (Invitrogen, Karlsruhe, Germany), 1.5mM MgCl_2_, 0.1mM dATP, 0.1mM dCTP, 0.1mM dGTP, 0.065mM dTTP, 0.035mM digoxigenin-11-dUTP (Roche Diagnostics), 10pmol primer, 5U Taq DNA Polymerase (Life Technologies, Darmstadt, Germany) and 50ng of human or chicken genomic DNA in a total volume of 50μl. The following primers were used: ALU-F 5`-CGAGGCGGGTGGATCATGAGGT-3`, ALU-R 5`-TTTTTTGAGACGGAGTCTCGC-3`CR1-F 5´-TCAGCCTGGAGAAGAGAAGG-3´, CR1-R 5´-CACCTCACCACTCTCCTGGT-3´. Paraffin sections were deparaffinized in Roti-Histol (Carl Roth GmbH), rehydrated in isopropanol and digested with proteinase K (Roche Diagnostics) diluted in PBS (5μg/ml) for 15 min at 37°C. After washing in PBS, sections were treated with 0.25% acetic acid containing 0.1M triethanolamine (pH 8.0) for 10 min and prehybridized for 1h at 42°C for ALU hybridization and 37°C for CR1 hybridization in hybridization buffer containing 4×SSC, 50% deionized formamide, 1×Denhardt’s solution, 5% dextrane sulfate and 100μg/ml salmon sperm DNA. Hybridization buffer was replaced by fresh buffer containing 0.2ng/μl digoxigenin-labeled ALU probe or 1ng/μl CR1 probe before target DNA and probe were denatured for 5min at 95°C. Hybridization was carried out for 16h at 42°C for ALU and at 37°C for CR1 in a wet chamber. Slides were washed twice for 5min in 2×SSC at room temperature and twice for 10 min at 42°C/37°C in 0.1×SSC. Signals were detected by immunohistochemistry using anti-Digoxigenin alkaline phosphatase-conjugated Fab fragments (Roche Diagnostics) and NBT/BCIP (Linaris, Dossenheim, Germany) as substrate. Sections were counterstained with methyl green (Linaris).

### Immunohistochemistry

Formalin fixed paraffin embedded osteosarcoma tissue sections and CAM assay xenografts were deparaffinized in Roti-Histol (Carl Roth GmbH) and rehydrated in isopropanol. The use of patient tissue was approved by the ethics committee of the University of Heidelberg (S-343/2015). Antigen retrieval was performed by incubation in citrate buffer pH 6 (Dako, Hamburg, Germany) for 20min at 90°C. Slides were blocked for 1h at 20°C in 5% bovine serum albumin (BSA), washed in PBS and incubated with primary antibodies diluted in 1% BSA for 16h at 4°C. The following antibodies were used at the indicated dilutions. RUNX2 (1:200) (Santa Cruz Heidelberg Germany), SPARC (1:100) (Santa Cruz), BMP-4 (1:100) (ProteinTech, Manchester, United Kingdom), PRIM1 (1:100) (ProteinTech) and CD34 (1:100) (Biorad, München, Germany). Signals were detected using a BrightVision +Poly-AP-anti Ms/Rb IgG kit (VWR International GmbH, Darmstadt, Germany) in combination with the alkaline phosphate substrate ImmPACT Vector Red (Linaris) according to the manufacturer´s instructions. Samples were counterstained with Mayers hematoxylin and mounted using Neo-Mount (Merck-Millipore, Darmstadt, Germany).

### Lens culinaris agglutinin staining

Visualization of chicken blood vessels was performed on 5μm paraffin sections of tumor xenografts. Samples were deparaffinized in Roti-Histol (Carl-Roth GmbH, Karlsruhe, Germany), rehydrated in isopropanol (100%, 96%, 70% and 50%, 5min each) and equilibrated in PBS. Sections were blocked with 5% BSA in PBS for 15 min at room temperature, washed in PBS and incubated for 30min in PBS supplemented with 5μg/ml biotinylated Lens culinaris agglutinin (Linaris). Sections were washed two times in PBST (PBS supplemented with 0.5% Tween 20), once in PBS and incubated for 30 min in AB reagent (Avidin D / biotinylated alkaline phosphatase) (Linaris). Sections were washed again as described above and incubated in ImmPACT Vector Red (Linaris) alkaline phosphatase substrate for 20 min. Section were washed in distilled H_2_O, counterstained with Methyl Green (Linaris) and mounted using Neomount (Merck-Millipore).

### Chick chorioallantois membrane assay

Fertilized white Leghorn chicken eggs were delivered by a local ecological hatchery (Geflügelzucht Hockenberger, Eppingen, Germany). Upon delivery eggs were cleaned with dry paper towels and sterile water. Eggs were incubated in an upright position with the pointed side facing downward at a humidity of 70%, a temperature of 37.8 C° and permanent agitation. This time point was designated as embryonic development day 0 (EDD 0). At EDD4, eggs were prepared for transplantation by horizontal positioning of the eggs and removal of 3 ml albumen at the wider end of the egg with a 20 gauge needle. This step was performed using diaphanoscopy allowing easy localization of the embryo and the yolk sac. A Leukosilk tape was applied on the upper side of the egg before the chorioallantoic membrane was exposed by cutting a window of approximately 1.5 cm diameter into the eggshell. This window was not completely removed in order to close and reseal it with Leukosilk for further incubation. If not otherwise stated transplantation of tumor cells was performed at EDD 9. The window was opened again and a sterile silicone ring (9mm inner diameter) was placed onto the CAM. The CAM area within this ring was gently lacerated using a 30 gauge needle before the cells were inoculated. Cells were mixed with different matrices and transplanted either as pellet or as cell suspension. Cell pellets were formed 24h before transplantation by mixing cells and matrix and pipetting 40μl drops onto the surface of a petri dish. Polymerization of the matrix material occurred within 30min at 37C°. The pellets were then covered with DMEM medium and stored at 37C° until transplantation. Cell suspensions were directly applied onto the CAM after mixing cells and matrix material. As matrices we used Matrigel (Corning GmbH, Kaiserslautern, Germany), Geltrex, a growth factor reduced variant of Matrigel (Thermo Fisher Scientific, Darmstadt, Germany), Cultrex BME Type 3, a matrigel equivalent (AMS Biotechnology, Frankfurt, Germany) and collagen type I (AMS Biotechnology). The acidic Collagen type I solution was neutralized with NaOH and used at a final concentration of 0.8mg/ml. All other matrices were thawed at 4C° and mixed with an equal volume of cells resuspended in DMEM medium. If not otherwise stated 1 x 10^6^ cells in 20μl medium were mixed with 20μl matrix material and transplanted onto the CAM. Seven days post transplantation at EDD16 the xenograft tumors were resected after humane euthanasia of the chick embryo by injection of 50μl of the pentobarbital Narcoren into the chicken vasculature. Embryos that died before EDD 16 were excluded from the study. The volumes of the excised tumors were estimated using the following formula: volume = 4/3×pi×r3 (r = 1/2×√ of diameter 1×diameter 2) [10]. Tumor take rates were calculated as: number of eggs with tumors *100 / number of eggs with vital embryos. All experiments were conducted at least in duplicates by the investigators JF and HS, primarily simultaneously. Due to high variabilities in the number of non-fertilized eggs, the development and viability of the embryos before EDD9, and variable tumor take rates and viability between EDD9 and EDD16, data were pooled and evaluated as one single experiment to ensure sufficient group sizes for the primary objectives (viability, tumor take rate and tumor volume).

### Data analysis and statistics

Statistical analyses were conducted using the SPSS software (IBM, Armonk, USA). Comparisons of two groups were performed by the Mann–Whitney U test. P-values below 0.01 were considered statistically significant. Reported p-values are two-sided.

## Results

### Ethanol treatment, resealing of the egg window and diaphanoscopy significantly influence viability of chicken embryos

**Fig 1:**
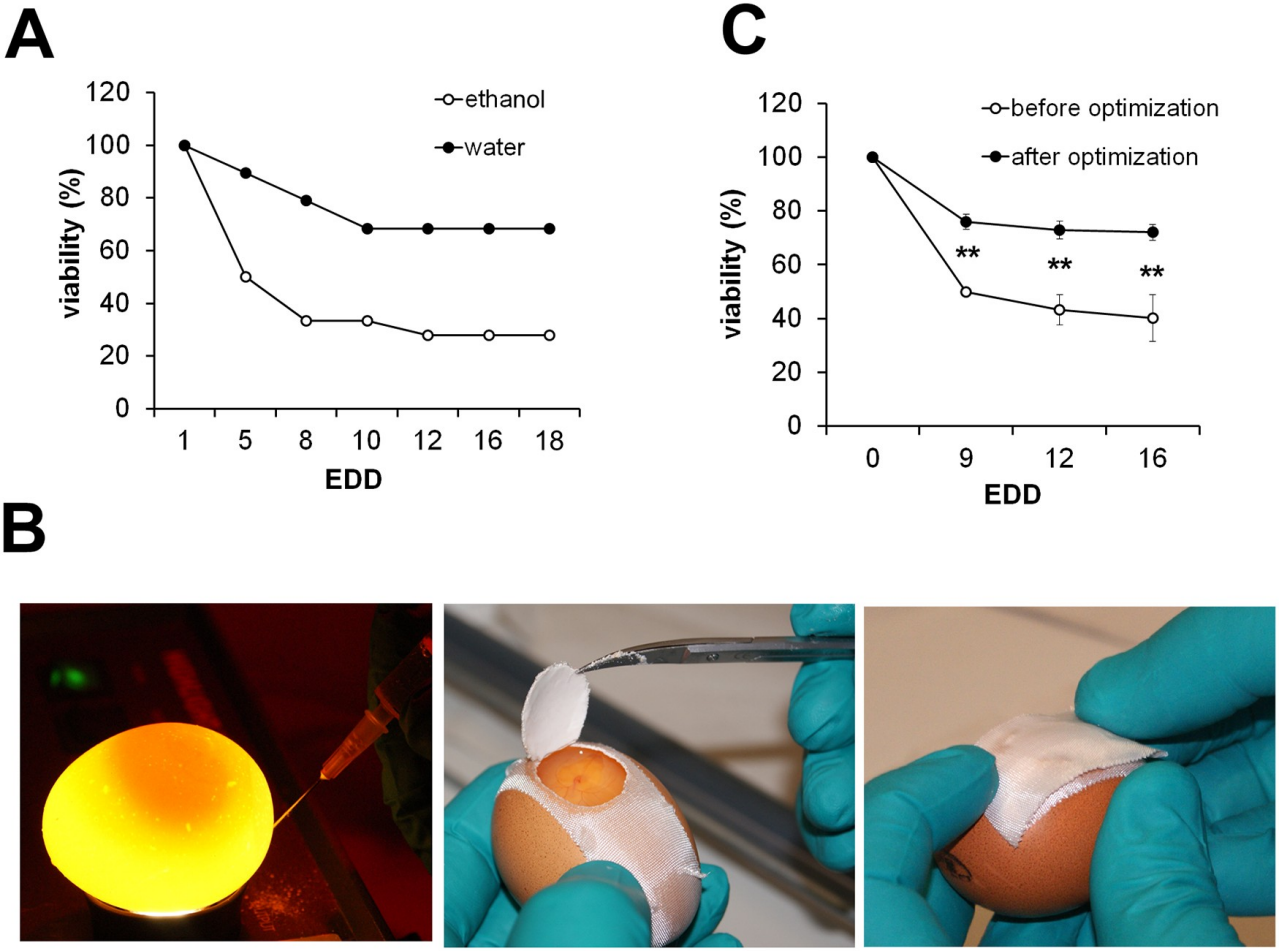
Cleaning, albumen removal and windowing of the eggs. A) Upon delivery eggs were cleaned either with ethanol or water (n = 20 each). Viability of the embryos was checked at the indicated time points (EDD = embryonic development day). B) Removal of albumen using diaphanoscopy, windowing of the eggs and resealing for further incubation. C) Viability before (n = 50) and after (n = 78) implementation of the described optimization steps (** p<0.01).

Unless otherwise stated all experiments carried out during the optimization of the CAM assay protocol were performed using the osteosarcoma cell line MNNG-HOS that has already been shown to produce tumors in CAM assays. Frequently reported problems during the application of CAM assays are reduced viability of the developing chicken embryos and contamination of the eggs. We assumed commonly applied 70% ethanol or other disinfectants on the eggshell to considerably influence the viability. To test this hypothesis, we analyzed the viability of chicken embryos after treatment of the eggs with ethanol compared to eggs treated with distilled water immediately after delivery. Viability was monitored from EDD 1 to EDD 18. Ethanol treatment significantly reduced viability, especially within the first five days to approximately 30% compared to 70% in the sterile water-treated group (Fig 1A). Notably, omitting the ethanol treatment did not lead to an increased rate of contamination. To further minimize the risk of contamination we did not completely remove the excised eggshell during windowing in order to be able to reseal the egg for further incubation (Fig 1B middle and right). To avoid injury of the embryo or the yolk sac during removal of albumen, a necessary step to avoid attachment of the CAM to the eggshell, we used diaphanoscopy allowing easy localization of the embryo and the yolk sac (Fig 1B left). In combination, omission of ethanol treatment, usage of diaphanoscopy and resealing of the window significantly increased the overall viability from initially 30%-40% to >80% (Fig 1C).

### Use of growth factor containing matrices increases tumor take rates and volumes

**Fig 2:**
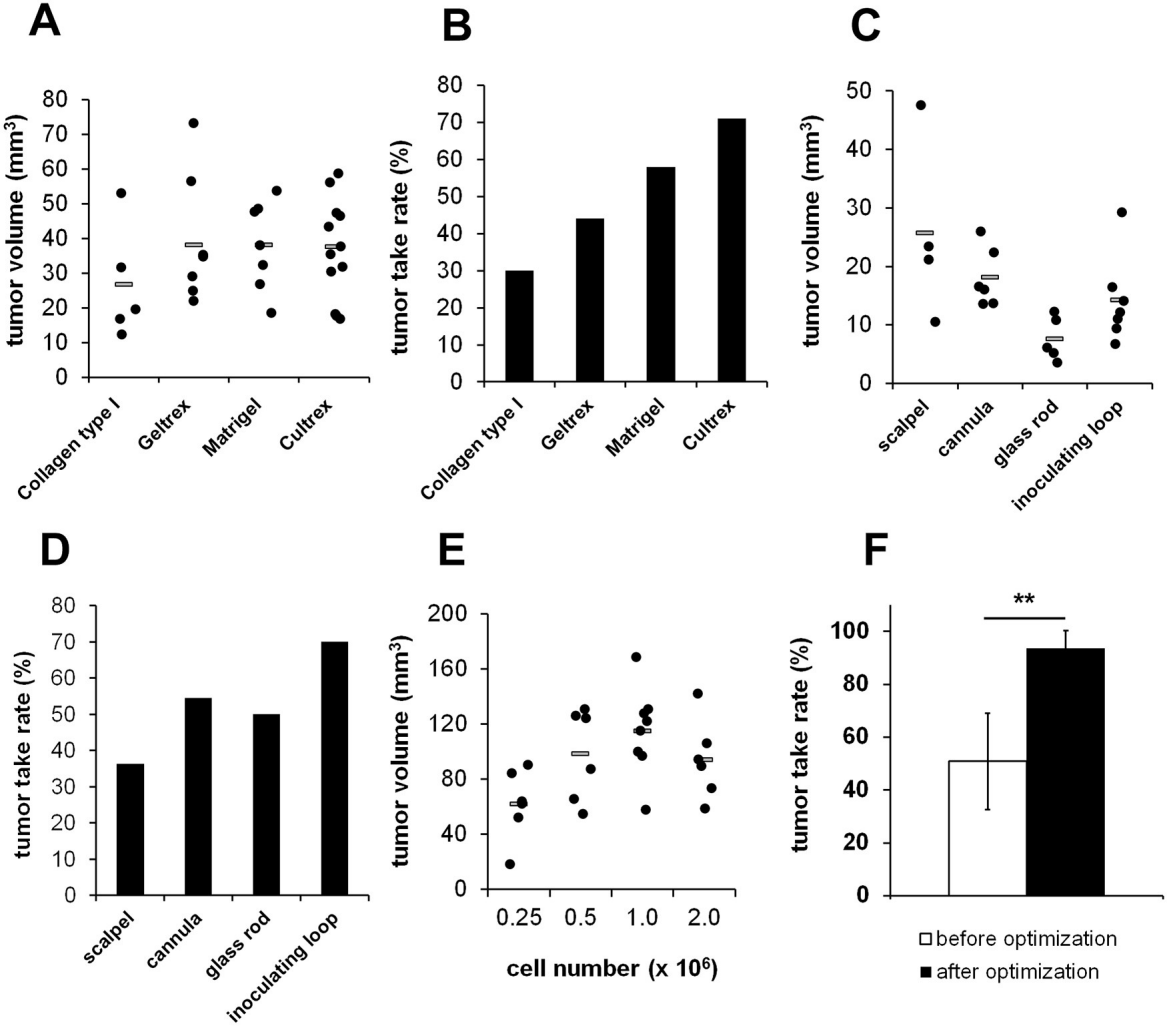
Effects of the cell matrix, the type of CAM pretreatment and the cell number on tumor take rates and tumor volumes. A) Tumor volumes achieved after transplantation of 1x10^6^ MNNG-HOS cells suspended in different matrices (n = 20 each). B) Calculated tumor take rates of MNNG-HOS cells suspended in different matrices. C) Influence of CAM pretreatment on the tumor volumes (n = 10 each). D) Calculated tumor take rates after different pretreatments. E) Influence of the number of transplanted MNNG-HOS cells on tumor volumes. F) Overall tumor take rates before (n = 25) and after (n = 78) implementation of the described optimization steps (** p<0.01).

We next aimed to further improve the protocol with regard to reproducibility, reliability, maximized tumor growth and tumor take rates. To ensure that tumor cells stay within a defined area on the CAM during inoculation, cells are immobilized within an extracellular matrix. We compared four different matrices with variable amounts of growth factors derived from different manufacturers. Collagen type I was used without any supplements and growth factors along with three variants of Matrigel, an extracellular matrix from the Engelbreth-Holm-Swarm mouse sarcoma. Matrigel was applied as growth factor containing and growth factor reduced variant termed Geltrex. In addition, a Matrigel equivalent from a different supplier termed Cultrex BME Type 3 was tested. While the volumes of the tumors that developed in the different Matrigel variants were comparable, tumor growth was markedly reduced when the growth factor free Collagen type I was used as matrix. Tumor take rates were considerably lower when growth factor free or reduced matrices were used compared to the growth factor containing matrices Matrigel and Cultrex BME Type 3 (Fig 2A and 2B).

### Gentle laceration of the CAM using a cannula and the inoculation of 1x10^6^ cells provides best results concerning tumor take rates and volumes

The rich vascular system of the CAM develops within the intermediate mesodermal layer that is located between the outer chorionic epithelium and the inner allantoic epithelium. To facilitate tumor engraftment and neovascularization the outer epithelium has to be partially removed by gentle laceration. We compared different techniques for the removal of the epithelium layer including inoculating loops used for bacterial cultivation, glass rods, a 30 gauge cannula and scalpels. The resulting tumor volumes were highest when a scalpel was used to scratch the CAM surface. However, we also observed a distinct reduction of the tumor take rate, probably due to injuries of the blood vessels and excessive hemorrhages. The best results concerning tumor volume and tumor take rate were observed when a cannula was used (Fig 2C and 2D). To analyze the impact of the initial cell number on the resulting tumor volumes we inoculated different amounts of tumor cells onto the CAM and observed a steady increase of the tumor volumes up to 1x10^6^ cells. At least for MNNG-HOS cells a further increase of the cell number had no positive effect on the tumor volumes (Fig 2D). Together, the use of growth factor containing matrigel as matrix, pretreatment of the CAM with a 30 gauge cannula and the use of 1x10^6^ cells as optimal cell number increased the overall tumor take rate from initially 51% to 94% (Fig 2F).

### Inoculation of a cell suspension is superior to premade cell pellets

**Fig 3:**
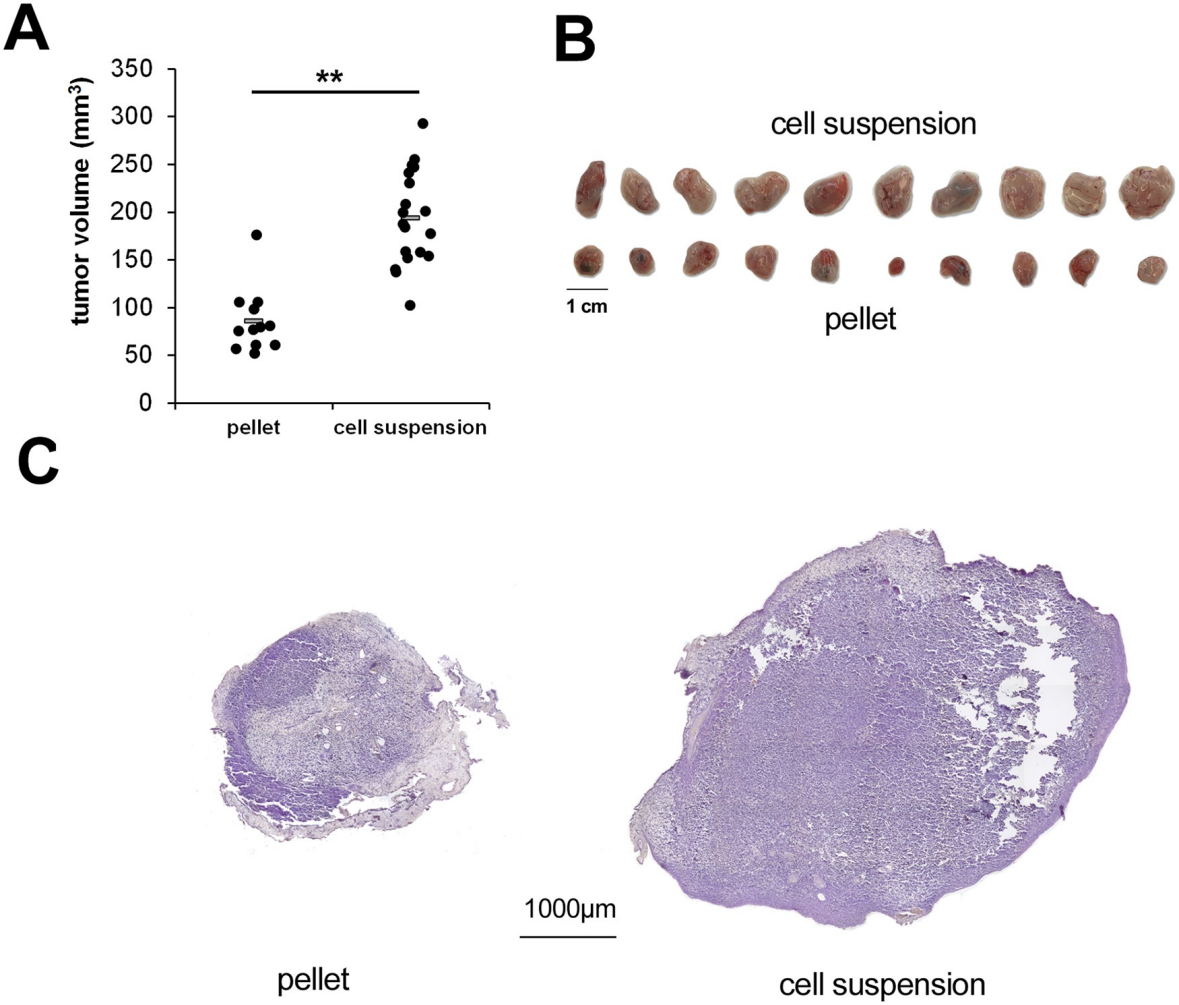
Increased tumor volumes after inoculation of cell suspensions compared to premade cell pellets. A) Calculated tumor volumes after transplantation of MNNG-HOS cells as cell pellets or cell suspension (n = 20 each). B) Photographs of resected xenografts C) Representative hematoxilin stainings of xenografts derived by transplantation of cell pellets or cell suspensions (** p<0.01).

To further increase the tumor volumes, we compared two types of cell inoculation procedures. One group was transplanted as premade cell pellets that were prepared by mixing the cells and matrix 24h before transplantation. In the other group the cells were inoculated as cell suspension directly after mixing with the matrix. Transplantation of cell suspensions resulted in a significant increase of tumor volumes, most likely due to the increased contact zone of tumor cells and CAM (Fig 3A–3C).

### Transplantation on EDD 9 and tumor resection on EDD 16 provides best results concerning tumor take rates and volumes

**Fig 4:**
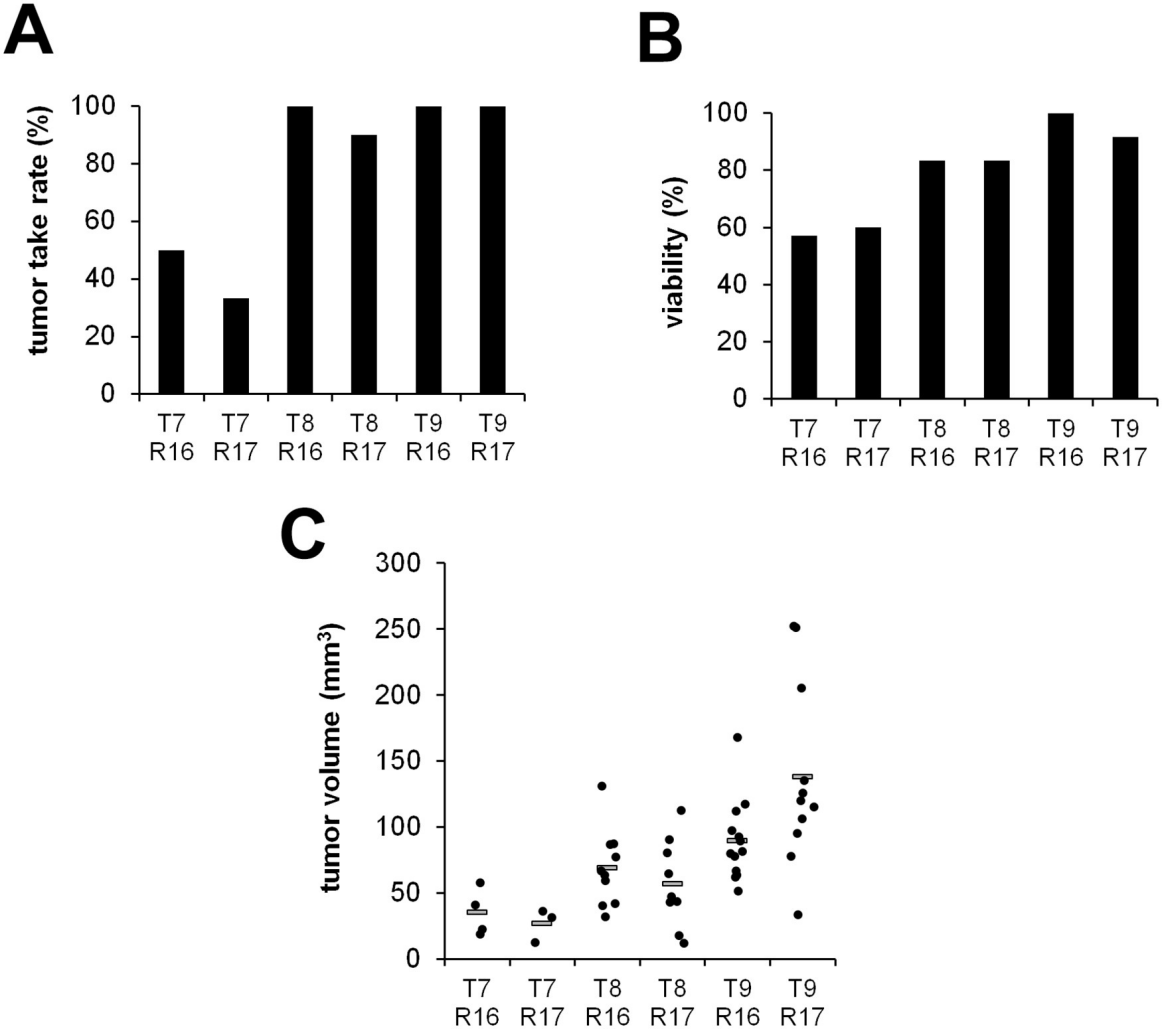
Determination of the optimal time points for cell transplantation and tumor resection. A) Tumor take rates achieved at different transplantation (T) and resection (R) time points. T7R16 (n = 14), T7R17 (n = 15), T8R16 (n = 12), T8R17 (n = 12), T9R16 (n = 13) and T9R17 (n = 12). B) Influence of these transplantation and resection time points on the viability of the embryos. C) Tumor volumes achieved at different transplantation and resection time points. (T = transplantation, R = resection).

We next investigated the influence of the length of the incubation period defined by the time of tumor cell transplantation and the time of tumor resection on tumor volumes and tumor take rates. Tumor take rates were primarily influenced by the time of transplantation. While cells transplanted on EDD 8 or EDD 9 developed solid tumors in 90% - 100% of all cases, transplantation on EDD 7 reduced tumor take rates to 33% - 50% (Fig 4A). Similarly, viability of the chicken embryos and the volumes of the developing tumors were reduced at earlier transplantation time points. Transplantation on EDD 9 and resection on EDD 16 turned out to provide optimal conditions (Fig 4B and 4C).

### Successful application of the established protocol on seven osteosarcoma cell lines

**Fig 5:**
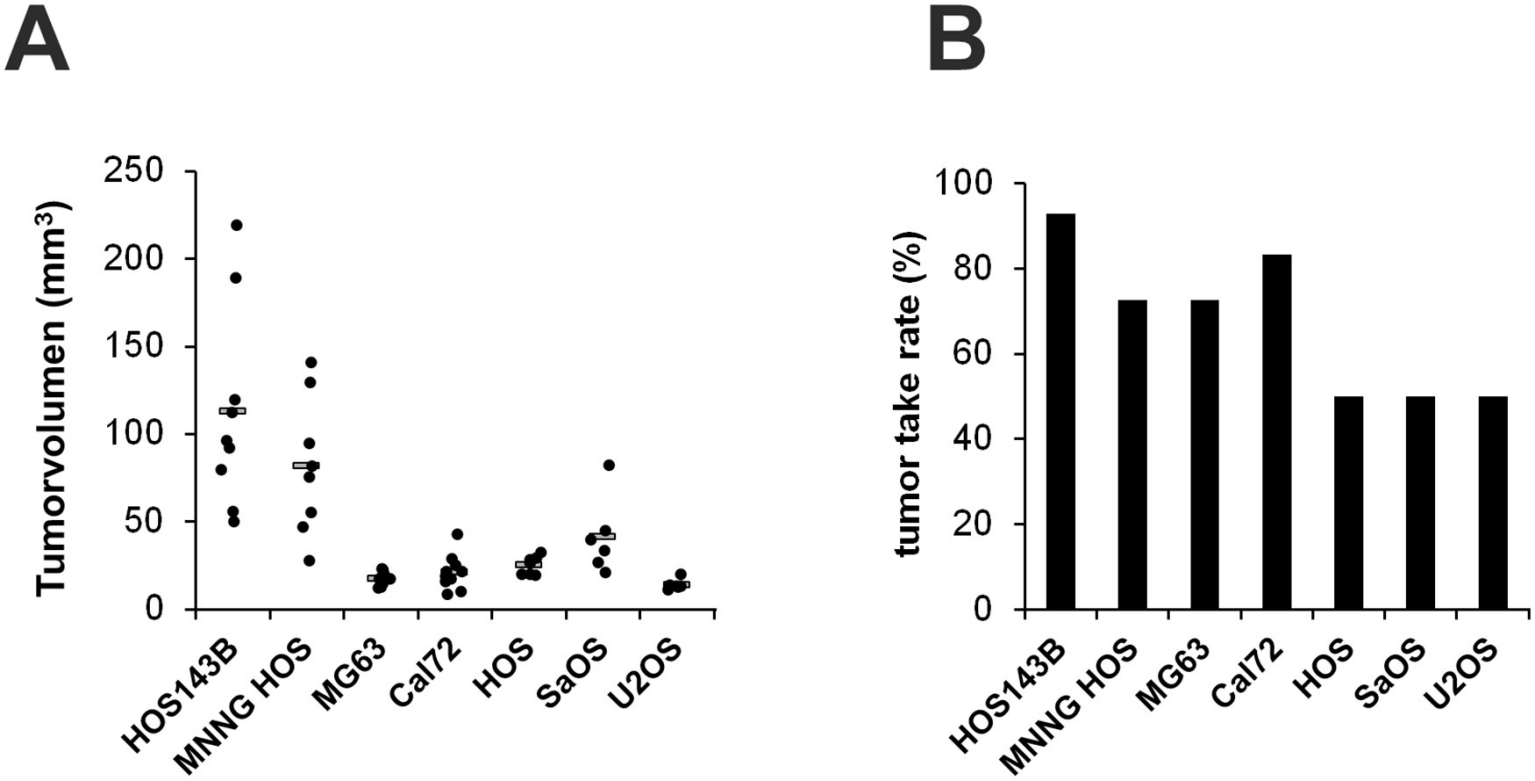
Suitability of the established CAM assay protocol for the analysis of osteosarcoma cell lines. A) Tumor volumes of xenografts derived after transplantation of the following osteosarcoma cell lines: HOS143B (n = 14). MNNG HOS (n = 16), MG 63 (n = 14), Cal72 (n = 14), HOS (n = 16), SaOS (n = 16) and U2OS (n = 14). B) Tumor take rates after transplantation of these osteosarcoma cell lines.

After optimization of the different CAM assay parameters with the osteosarcoma cell line MNNG-HOS we tested the established protocol for its usability as reliable xenograft model with further six osteosarcoma cell lines. Notably, all tested cell lines developed solid tumors with varying tumor take rates and tumor volumes (Fig 5A and 5B).

### Characterization of CAM assay xenografts

**Fig 6:**
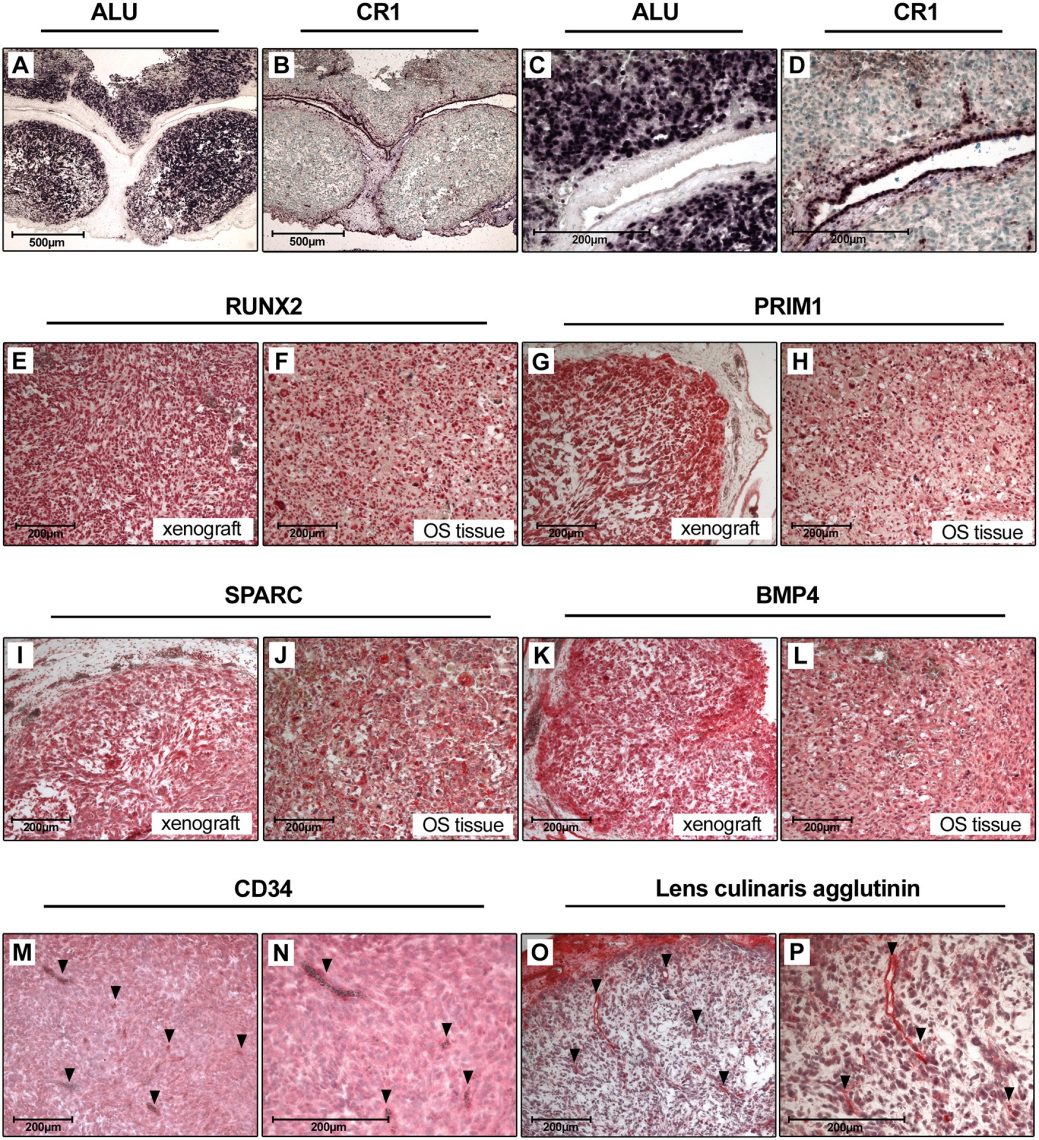
Characterization of CAM assay derived xenografts. A) and C) ALU in situ hybridization and B) and D) CR1 in situ hybridization of CAM xenografts. Nuclei of human (ALU) and chicken (CR1) cells are stained dark purple. Sections were counterstained with methyl green (Magnification in A and B is 50-fold, in C and D 200-fold). E-L) Immunohistochemical stainings of RUNX2, PRIM1, SPARC and BMP4 in CAM xenografts and osteosarcoma tissue (Magnification 100-fold). M) and N) CD34 staining of CAM xenografts. Vessels are indicated by arrows. O) and P) Lens culinaris agglutinin staining of CAM xenografts. Vessels are indicated by arrows (Magnification in M and O is 100-fold, in N and P 200-fold).

To verify that the isolated CAM assay xenografts developed from the transplanted human osteosarcoma cells we performed a human ALU repeat specific *in situ* hybridization. As a control, chicken cells were visualized using a chicken CR1 repeat specific *in situ* hybridization. Hereby we could demonstrate that the tumor mass of the xenografts is composed of human cells with a few infiltrating chicken cells, chicken vessels and the surrounding CAM (Fig 6A–6D). For further characterization of the xenografts we analyzed the expression of osteogenic marker proteins frequently overexpressed in osteosarcoma tissue partially as a consequence of chromosomal copy number gains [21]. These markers include RUNX2 (Runt-related transcription factor 2), SPARC (Secreted protein acidic and rich in cysteine or osteonectin), BMP-4 (Bone morphogenetic protein 4) and PRIM1 (DNA primase subunit 1). Protein expression of these markers in CAM assay derived xenografts were comparable to those in osteosarcoma biopsy tissues as shown by immunohistochemical analysis (Fig 6E–6L).

For further studies we aimed to detect and quantify intratumoral vessels of osteosarcoma xenografts. Several tested human CD31 antibodies did not cross react with chicken vessels. Applying chicken-specific CD34 immunohistochemistry allowed specific vessel detection, yet we were not able to fully control background staining in the CAM osteosarcoma xenografts, despite the application of several antigen retrieval and blocking techniques. As an alternative, we used biotinylated lens culinaris agglutinin, a lectin that specifically binds to chicken endothelial cells of arteries, veins and capillaries [22]. Using this approach, we could specifically stain vessels derived from chicken endothelium within the tumor xenografts without relevant background staining (Fig 6M–6P).

### Validation of the optimized protocol

**Fig 7:**
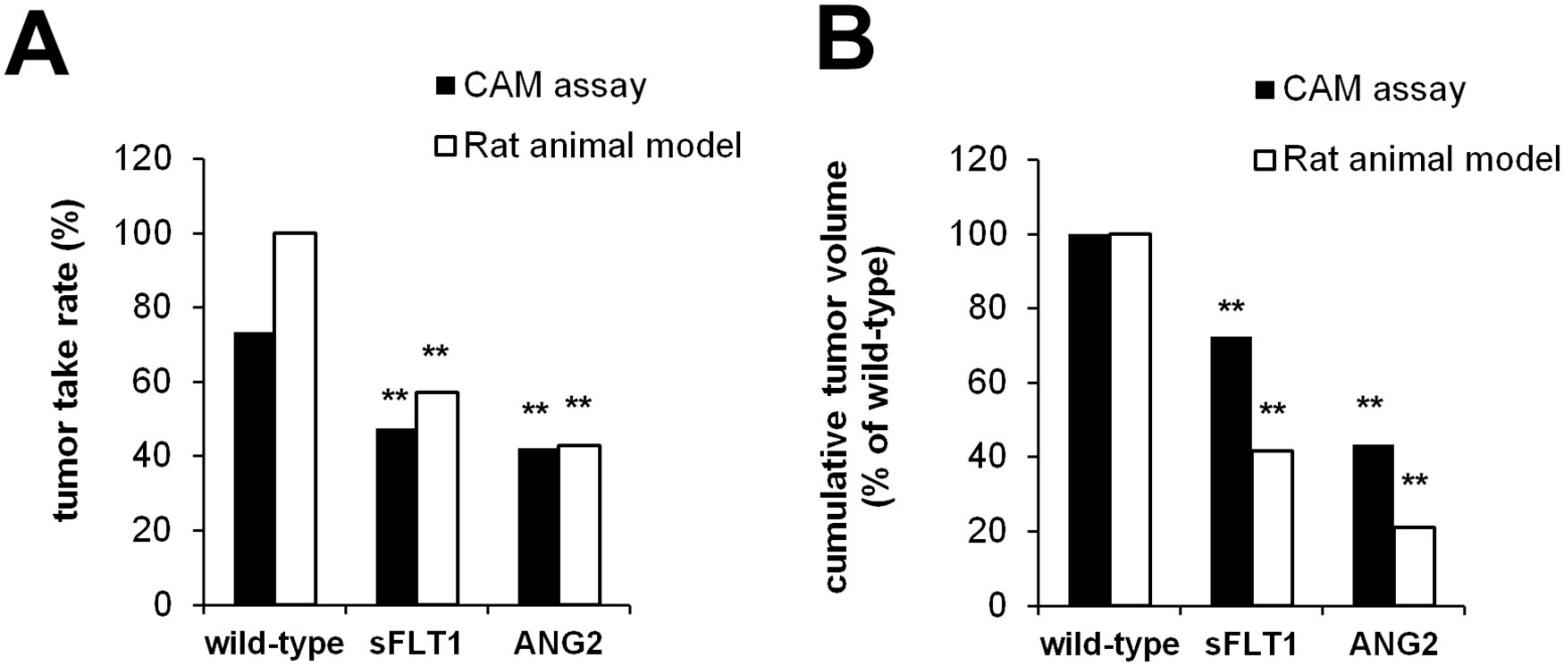
Comparison of the established CAM assay with a rat animal xenograft model. Rat osteosarcoma cells UMR-106 were stable transfected with sFLT1 and ANG2, respectively, and transplanted to chicken eggs (wild-type n = 24, sFLT1 n = 23, ANG2 n = 23) or subcutaneously injected into the lower leg of rats (n = 6 each group). A) Tumor take rates observed after transplantation of wild-type and transfected cells. B) Cumulative tumor volumes of wild-type and transfected cells (** p<0.01 compared to wild-type cells).

In order to validate our optimized osteosarcoma CAM-Assay protocol with regard to its suitability to substitute animal experiments according to Russel and Burch 3Rs, we compared outcome parameters obtained using the established CAM assay protocol with data obtained using a rat animal model. The rat osteosarcoma cell line UMR-106 was stable transfected with the angiogenesis modulating genes sFLT1 (soluble fms-like tyrosine kinase-1) or ANG2 (angiopoetin-2). In vitro doubling time of wild-type-, sFLT1 and ANG2-overexpressing UMR-10^6^ cells were comparable. Wild-type cells and sFLT1 and ANG2-overexpressing cells were transplanted to the CAM or were subcutaneously injected into the lower leg of rats. Stable overexpression of sFLT1 and ANG2 significantly reduced tumor take rates and the volumes of the resulting tumors. The observed effects were highly comparable between the CAM assay and the rat osteosarcoma animal experiment (Fig 7A and 7B).

## Discussion

*In vivo* models are indispensable tools for the study of osteosarcoma biology and the identification of new therapeutic targets. Especially the heterogeneity of this tumor, the interaction with the tumor microenvironment and the intratumoral vascularization are not sufficiently taken into account when *in vitro* assays are applied. In our effort to reduce or even replace animal experiments we aimed to investigate the suitability, reliability and reproducibility of the chicken chorioallantoic membrane assay as an *in vivo* tumor model for osteosarcoma research. Although the CAM assay is widely used to study angiogenesis and the biology of various types of tumors little information is available about the use of this assay for osteosarcoma research. The ability of the osteosarcoma cell line Saos-2 to induce new blood vessel formation by upregulation of angiogenic growth factors like VEGF165, FGF2, MMP2 and MMP9 has already been shown. However this study only focused on the early phase of tumor growth within the first 96 hours [23]. Another study also demonstrated the formation of solid tumors and the induction of an angiogenic response after transplantation of different osteosarcoma cell lines onto the CAM. However, only 3 out of 8 cells lines consistently formed vascularized tumors and the mortality rate of the embryos was very high ranging from 18% - 70% [18]. Interestingly, tumor grafts derived from sarcoma patients have been shown to retain tumor morphology, viability, and invasion potential in the chick chorioallantoic membrane model [24].

In order to optimize the existing CAM assay protocols for a reliable application in osteosarcoma research we initially aimed to reduce the mortality rate of the chicken embryos. As also suggested by Sys et al. [19], we asumed that the excessive use of ethanol or other disinfectants strongly influences the viability of the embryos due to the permeability of the eggshell and might even be unnecessary. In fact, omitting disinfectants significantly reduced the mortality rate without any increase of contaminations. A further improvement of the survival rate of the embryos could be achieved by minimizing the risk of damge during the opening of the eggs and the removal of albumen by using diaphanoscopy during these steps.

Further major outcome parameters that had to be improved were the tumor take rate and the achieved tumor volumes. Grafting of tumor cells and the formation of solid tumors strongly depends on the accessibility of the chicken vasculature. It has been shown that transplanted tumors enter an avascular phase of about 72 h before vessels begin to penetrate the tumor, initiating the vascular phase that is characterized by a rapid growth rate. Tumors >1mm diameter that do not reach the vascular phase will undergo necrosis [25]. In this respect, pretreatment of the CAM plays an important role to facilitate tumor engraftment and neovascularization. We observed that mild treatments using a glass rod or inoculating loops were less effective than scratching the upper epithelium layer of the CAM with a cannula. We assume that the improved accessibility of the vasculature shortens the avascular phase leading to increased tumor volumes. A harsh treatment on the other side induced a worse tumor take rate due to injuries of the blood vessels and excessive hemorrhages. Our observation of increased tumor take rates and tumor volumes after transplantation of tumor cells in growth factor containing matrices might also be explained by an enhanced survival of tumor cells during the avascular phase. Finally, the mode of cell inoculation turned out to have a significant impact on tumor volumes in osteosarcoma. When cells were transplanted as premade cell pellets the volumes of the resulting tumors were significantly lower than those that formed after inoculation of cell suspensions. Most likely the increased contact area between suspended cells and CAM accelerates cell-cell interactions and facilitates the penetration of new blood vessels, thus, extending the vascular growth phase.

In summary, only the systematic adaptation of various parameters provided a reliable and reproducible *in vivo* model system for the analysis of osteosarcoma including the analysis of intratumoral vascularization. Notably, the capacity of the developed protocol could be confirmed by the successful generation of solid tumor from all eight osteosarcoma cell lines tested in this study. Furthermore, the validation of our optimized OS-CAM-assay against a conventional *in vivo* osteosarcoma rat model allowed an exact reproduction of changes in the major outcome parameters after inoculation of genetically altered osteosarcoma cells. These observations confirm the capability of this *in vivo* model to at least partially substitute animal experiments in osteosarcoma research.

## Conclusions

The CAM assay can bridge the gap between *in vitro* cell culture and *in vivo* animal experiments. As an *in vivo* model for osteosarcoma research it helps to investigate tumor properties and cellular mechanisms including tumor microenvironment, may speed up preclinical data collection and simplifies research on potential new agents towards personalized medicine. The reasonable use of this model provides a refinement by minimizing pain and suffering of animals and supports a considerable reduction and/or replacement of animal experiments in accordance with Russell’s and Burch’s “Principles of Humane Experimental Technique”.

## Supporting information

S1 Supporting Information ARRIVE checklist. (PDF)

S2 Supporting Information Minimal data sets of the experiments shown in Figs 1, 2, 3, 4, 5 and 7. (XLSX)

## Abbreviations

ANG2: angiopoetin-2
CAM: Chorioallantoic membrane
DMEM: Dulbecco’s Modified Eagle Medium
ECM: extracellular matrix
EDD: embryonic development day
FCS: fetal calf serum
sFLT1: soluble fms-like tyrosine kinase-1
